# MicroRNA-425-5p Expression Affects BRAF/RAS/MAPK Pathways In Colorectal Cancers

**DOI:** 10.7150/ijms.35269

**Published:** 2019-10-11

**Authors:** Andrea Angius, Giovanna Pira, Antonio Mario Scanu, Paolo Uva, Giovanni Sotgiu, Laura Saderi, Alessandra Manca, Caterina Serra, Elena Uleri, Claudia Piu, Maurizio Caocci, Gabriele Ibba, Angelo Zinellu, Maria Rosaria Cesaraccio, Francesca Sanges, Maria Rosaria Muroni, Antonina Dolei, Paolo Cossu-Rocca, Maria Rosaria De Miglio

**Affiliations:** 1 Istituto di Ricerca Genetica e Biomedica (IRGB), CNR, Cittadella Universitaria di Cagliari, 09042 Monserrato (CA), Italy;; 2 Department of Biomedical Sciences, University of Sassari, Viale San Pietro 43-b, 07100 Sassari, Italy;; 3 Department of Medical, Surgical and Experimental Sciences, University of Sassari, Viale San Pietro 8, 07100 Sassari, Italy;; 4 CRS4, Science and Technology Park Polaris, Piscina Manna, 09010 Pula, CA, Italy;; 5 Department of Pathology, AOU Sassari, Via Matteotti 60, 07100 Sassari, Italy;; 6 Department of Prevention, Registro Tumori Provincia di Sassari, ASSL Sassari-ATS Sardegna, Via Rizzeddu 21, Sassari, Italy;; 7 Department of Diagnostic Services, “Giovanni Paolo II” Hospital, ASSL Olbia-ATS Sardegna, Via Bazzoni-Sircana, 07026 Olbia, Italy.

**Keywords:** Colorectal carcinoma, KRAS mutation, miR-425-5p expression levels, DICER1 gene, TNFRSF10B gene, PTEN gene.

## Abstract

Colorectal cancer (CRC) is a leading cause of cancer death worldwide and about 20% is metastatic at diagnosis and untreatable. The anti-EGFR therapy in metastatic patients is led by the presence of KRAS-mutations in tumor tissue. KRAS-wild-type CRC patients showed a positive response rate of about 70% to cetuximab or panitumumab combined with chemotherapy. MiRNAs are promising markers in oncology and could improve our knowledge on pathogenesis and drug resistance in CRC patients. This class of molecules represents an opportunity for the development of miRNA-based strategies to overcome the ineffectiveness of anti-EGFR therapy.

We performed an integrative analysis of miRNA expression profile between KRAS-mutated CRC and KRAS-wildtype CRC and paired normal colic tissue (NCT). We revealed an overexpression of miR-425-5p in KRAS-mutated CRC compared to KRAS-wild type CRC and NCT and demonstrated that miR-425-5p exerts regulatory effects on target genes involved in cellular proliferation, migration, invasion, apoptosis molecular networks. These epigenetic mechanisms could be responsible of the strong aggressiveness of KRAS-mutated CRC compared to KRAS-wildtype CRC. We proved that some miR-425-5p targeted genes are involved in EGFR tyrosine kinase inhibitor resistance pathway, suggesting that therapies based on miR-425-5p may have strong potential in targeting KRAS-driven CRC. Moreover, we demonstrated a role in the oncogenesis of miR-31-5p, miR-625-5p and miR-579 by comparing CRC versus NCT.

Our results underlined that miR-425-5p might act as an oncogene to participate in the pathogenesis of KRAS-mutated CRC and contribute to increase the aggressiveness of this subcategory of CRC, controlling a complex molecular network.

## Introduction

Colorectal cancer is the most frequent carcinoma and the third most common cause of cancer-related death worldwide 1. About 20% of CRC patients already have metastases at diagnosis, and metastatic CRC is not a treatable disease 2. KRAS oncogene regulates the downstream effectors activation of several pathways, such as BRAF/RAS/MAPK, PI3K/AKT, RalGDS/p38MAPK, etc., thus influencing normal cell physiology, neoplastic cell biology and therapeutic responses. Almost 40% of CRCs reported KRAS mutations that were predictive biomarkers of treatment efficacy and patient outcome 3. The KRAS mutations in exon 2 are related to more advanced tumors and unfavorable prognosis 4,5. The identification of KRAS mutations is a widely accepted molecular test considering targeted therapies in metastatic CRC 6,7. The presence of wild-type KRAS sequences ensures successful targeting by monoclonal antibodies (Cetuximab or Panitumumab) of the anti-EGFR axis. A better understanding of the tumor biology and predictive factors is crucial for the identification of new therapeutic targets in KRAS-mutated CRC patients.

MicroRNAs (miRNAs) are involved in the regulation of multiple signaling pathways (cell cycle regulation, proliferation, differentiation and apoptosis) 8,9, whose deregulation is involved in the development of tumors and could be potentially therapeutic targets 10*.* It is currently accepted that the miRNA expression profile shows high accuracy at classifying tumors 11. Specifically, miRNA expression level variations have identified between normal and neoplastic colorectal tissues. *In vitro* and in animal models, anti-cancer miRNA mimics inhibit CRC cancer cells proliferation, migration and induce apoptosis 12,13. Recent studies prove that several miRNAs are implicated in responding to chemotherapy 14 and that specific miRNAs have even shown prognostic potentials in CRC 15. MicroRNAs may be of critical importance for the diagnosis, treatment and prediction of outcomes in CRC patients.

Our study focuses on miRNAs expression profile in CRC after KRAS mutations screening. These two subcategories, which show different therapeutic perspectives, could be instrumental in providing further insights into the molecular mechanisms of tumorigenesis and identifying new molecular targets to improve the therapeutic strategies of KRAS mutated CRC patients.

## Material and Methods

### Patients and samples

The study was conducted according to the recommendations of the Helsinki Declaration and approved by the Bioethics Committee of the Azienda Sanitaria Locale Sassari, Italy (n. 2032/CE, 13/05/2014). All patients gave written informed consent for tissue banking and genetic analysis.

We screened one hundred and twenty anonymized and consecutive patients diagnosed with CRC that underwent surgical resection at the Surgery Unit of University of Sassari starting from June 2014 to December 2015. Forty-seven primary colorectal carcinoma and related NCT were enrolled in the present study, after the exclusion of patients who received neoadjuvant chemo and/or radiotherapy and showed multiple recurrence and/or CRC familiarity. Tissue samples were stored in RNAlater solution at -80°C. All tumors were critically assessed by a pathologist, and achieved a final diagnosis of CRC according to WHO criteria 16. All clinical-pathologic and follow-up data were available from medicals records for all CRC patients. The follow-up started at time of diagnosis (June 2014 - December 2015) and ended on 28 February 2018.

### DNA and RNA extraction

Genomic DNA was extracted from neoplastic tissue by using the QIAamp DNA Mini Kit (Qiagen, Hilden, Germany). Total RNA was extracted from neoplastic and non-neoplastic tissues by homogenizing 100 mg of tissue in 1 ml of Qiazol (Qiagen) and using miRNeasy Mini Kit (Qiagen) according to the manufacturer's instructions. DNA and RNA concentration and purity were assessed using the Nanodrop ND-1000 spectrophotometer (Thermo Fisher Scientific, Waltham, MA, USA). RNA underwent a Qubit-fluorometric quantification using Qubit® RNA BR Assay Kit (Thermo Fisher Scientific). The RNA integrity was assessed by the RNA Integrity Number (RIN) using the Agilent RNA 6000 Nano Kit on the BioAnalyzer 2100 (Agilent, Santa Clara, CA, USA).

### Mutation analysis

KRAS gene mutation analysis was performed on codons 12 and 13 of exon 2 and codons 59 and 61 of exon 3, which are known to harbor the most frequent and significant activating mutations for this gene 17, resulting in impaired intrinsic and GTPase-activating protein (GAP)-mediated GTP hydrolysis and leading to elevated levels of cellular RAS-GTP 18. Amplification of entire exon 2 and 3 were performed using the following sequence primers, respectively: forward-GTTTGTATTAAAAGGTACTGGTGGA reverse-ATCAAAGAATGGTCCTGCAC and forward-TCAAGTCCTTTGCCCATTTT reverse-ACCCACCTATAATGGTGAATATC. Gene sequencing analysis was executed as previously reported 19. Whereas, the same CRC samples were analyzed by RNAseq (unpublished observations from manuscript under review), the results obtained were screened for the presence of other variant mutations in the entire KRAS gene; especially, for mutations on codons 117 and 146 of the exon 4.

### Human miRNA card array and quantitative real-time PCR

The high-throughput miRNA expression profiling was first performed on eight pairs of CRC and NCT tissues from the same samples. These patients are part of the subsequent validation cohort. We used the TaqMan® Array Human MicroRNA Card A and B set v3.0 (Thermo Fisher Scientific), which enables analysis of a total of 754 human miRNA assays present in the miRBase version 18.0. The cards contain three endogenous controls (MammU6, RUN44, and RUN48) for relative quantization, of which only MammU6 was present in four replicates while the other two controls appeared just once, and an assay unrelated to any mammalian species, ath-miR-159a, as a negative control. Total RNAs (1000 ng) were converted to cDNAs using Megaplex™ RT Primers Human Pool A and B (Thermo Fisher Scientific), each Pool A and B contain a set of 377 stem-looped reverse transcriptional primers and 4 controls, and TaqMan® MicroRNA Reverse Transcription kit (Thermo Fisher Scientific). The reverse transcription mix included 1.07x Megaplex™ RT Primers Human Pool A or Pool B, 1.07x RT buffer, 0.65mM each of dNTPs, 3mM MgCl2, 75U/μl MultiScribe reverse transcriptase, and 2U/μl RNase inhibitor. The 7.5 μl reactions were incubated at the following conditions: 40 cycles at 16°C for 2 minutes, 42°C for 1 minute and at 50°C for 1 second, and 1 final cycle at 85°C for 5 minutes.

PCRs were performed using 450μl TaqMan® Universal PCR Master Mix, No AmpErase UNG (2X; Thermo Fisher Scientific), and 6 μl diluted pre-amplification product in a final volume of 900 μl. One hundred μl of the PCR mix were dispensed into each port of the TaqMan miRNA array, and then the fluidic cards was centrifuged and mechanically sealed. The 384-well format TaqMan Low Density Array (TLDA) arrays were run on an ABI 7900HT Fast Real-Time PCR system at the following conditions: 50°C for 2 minutes, 94.5°C for 1 minute, and 40 cycles at 97°C for 30 seconds and 59.7°C for 1 minute. RT-qPCR raw data were analyzed using SDS 2.4 and RQ Manager Software (Thermo Fisher Scientific).

The differential expression of significantly deregulated miRNAs (p-value < 0.05) was further validated by RT-qPCR in the entire dataset (47 CRC and 47 NCT) according to 20.

Briefly, the cDNA synthesis was performed as described above. The PCR reactions were carried out in final volumes of 10 μl using the Applied Biosystems 7900HT Fast Real-Time PCR System (Thermo Fisher Scientific). Reaction mix consisted of 54 ng of reverse-transcribed RNA, 1x TaqMan® Universal PCR Master Mix, 0.2 mM TaqMan® primer-probe mix (Thermo Fisher Scientific). An RT-negative control was included in each batch of reactions. Cycling conditions were: 10 minutes of denaturation at 95°C, 40 cycles at 95°C for 15 seconds and at 60°C for 1 minute. MiRNA U6 was used as reference for normalizing miRNA expression. All reactions were performed in triplicate.

### Identification of miRNA experimental gene targets

The target genes of CRC-related to differentially expressed miRNAs were predicted by seven algorithms: DianaMicroT_strict 21, miRanda-mirSVR_S_C 22, MirTarget2 23, picTar_chicken 24, PITA_Top 25, starBase 26 and TargetScan_v6.2 27. Experimentally validated targets were identified by literature and/or from miRecords 28 and mirTarBase v4.5 29 databases. Comparisons of target genes lists were performed with custom scripts using the computing environment R 30. Targets predicted by at least three of the seven algorithms or previously experimentally validated (i.e. reported in at least one database or in literature) were selected for subsequent analysis, in order to obtain improved results.

To inspect the function of the differentially expressed miRNAs, the target genes were submitted to Gene Ontology (GO) and KEGG pathway enrichment analysis using ToppCluster (https://toppcluster.cchmc.org/) 31. Terms with False Discovery Rate (FDR) corrected enrichment p-values <0.05 were considered. ToppCluster results were displayed using Cytoscape, which is an open source software used to build and display the miRNA-target gene network (http://www.cytoscape.org/) 32.

### Statistical analysis

Evaluation of the relative miRNA expression was calculated by the comparative cycle threshold method (2^-ΔΔCt^) 33 and the Ct values normalization by the quantile method.

The clustering based on expression profiles was carried out using an unsupervised hierarchical clustering, utilizing Pearson's correlation as a distance measure and average linkage as agglomerative algorithm. The statistical analysis of Microarray (SAM) analysis has identified miRNAs with statistically significant changes in expression profile. A FDR corrected p-value <0.05 was applied to identify statistically significant differences. All differential expression analyses were run in R using the *samr* package 34. Descriptive and inference statistics were carried out with the statistical software STATA version 15 (StatsCorp, Texas, US). A survival analysis was carried to assess the role played by the selected miRNAs. Logistic regression analyses were carried out to assess the relationship between miRNAs levels and clinical and epidemiological variables.

## Results

### KRAS mutational status

Mutational analysis of the KRAS gene has been carried out in all CRCs. KRAS somatic missense mutations were detected in 18 out of 47 CRCs (38.2%), whereas 29 out of 47 CRCs (61.8%) had no variants. In exon 2, our results identified variants on codon 12 in 14 out of 18 CRC (77.8%) (p.Gly12Asp in 9 tumors, p.Gly13Val in 3 tumors, p.Gly12Ala and p.Gly12Ser in one tumor, respectively), while mutations on codon 13 are present only in 3 out of 18 CRC (16.7%; p. Gly13Asp). Analysis of the exon 3 revealed the mutation p.Glu61Leu in a single CRC (5.5%). RNASeq analysis was in accordance with Sanger Sequencing for all CRC samples. Moreover, RNASeq data did not identify any mutation on exon 4.

### MiRNA expression profiles

**Figure 1:**
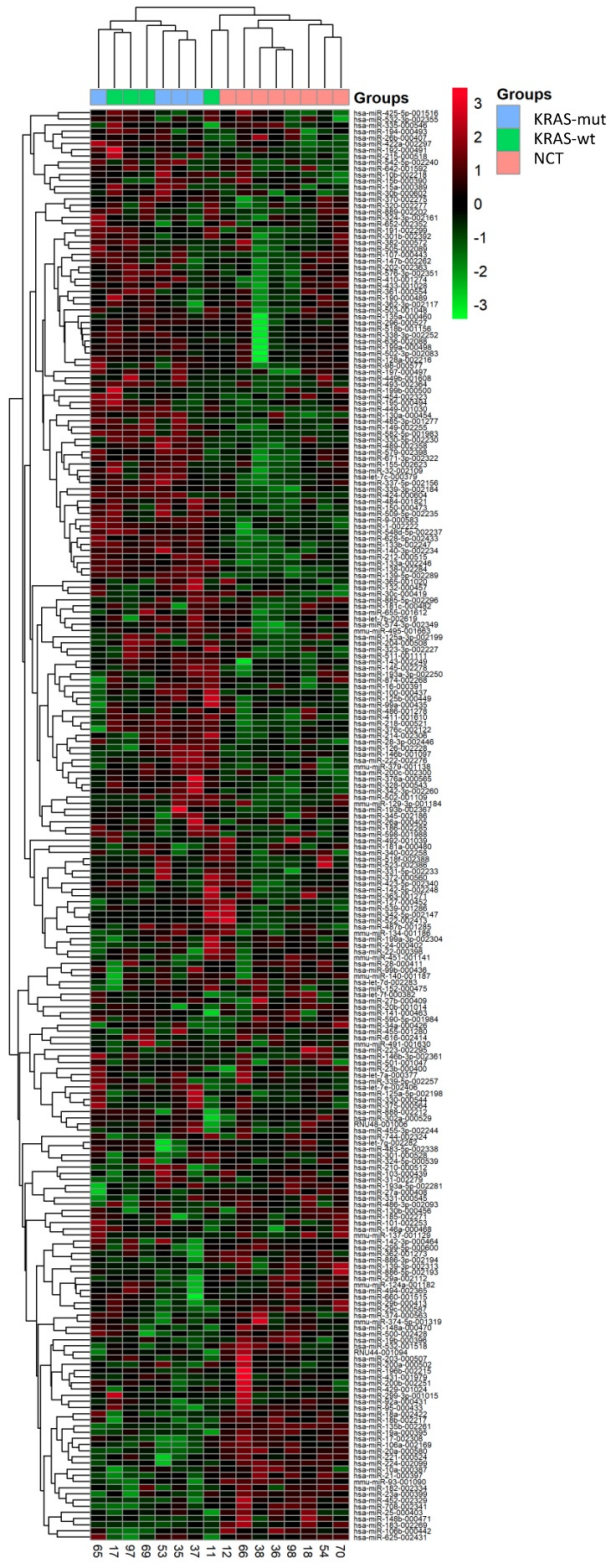
** A 220-miRNAs expression signature reveals changes between CRC with and without KRAS mutation and NCT**. Unsupervised hierarchical clustering analysis of CRC with KRAS mutation (blue), CRC with KRAS wildtype (green) and normal colon tissue (NCT; pink) was performed using 220 differentially expressed miRNAs. Dendrograms of clustering analysis for samples and miRNAs are displayed on the top and left, respectively, and depicts similarities in the miRNA expression profiles among the samples. The relative up and down regulation of miRNAs is indicated by red and light green, respectively. hsa; *Homo sapiens*. Endogenous control assays are present (mmu-miR and RNU).

We performed RT-qPCR data using TaqMan Low-Density Array (TLDA) in CRC and NCT. After normalization and removal of miRNAs that were not expressed in our cohort, miRNAs expression levels were used to perform unsupervised hierarchical clustering analysis, which allowed clear separation of healthy colon mucosa from tumors. Afterwards, the cancer tissues were divided in two subgroups: CRC with mutation in KRAS gene and CRC with wildtype KRAS gene (Figure 1). SAM analysis identified five miRNAs differentially expressed between KRAS-mutated CRC and KRAS-wildtype CRC and between KRAS-wildtype CRC and NCT. The RT-qPCR analysis performed on 754 miRNAs has allowed identifying deregulated miRNAs included only in TLDA Card A.

**Figure 2:**
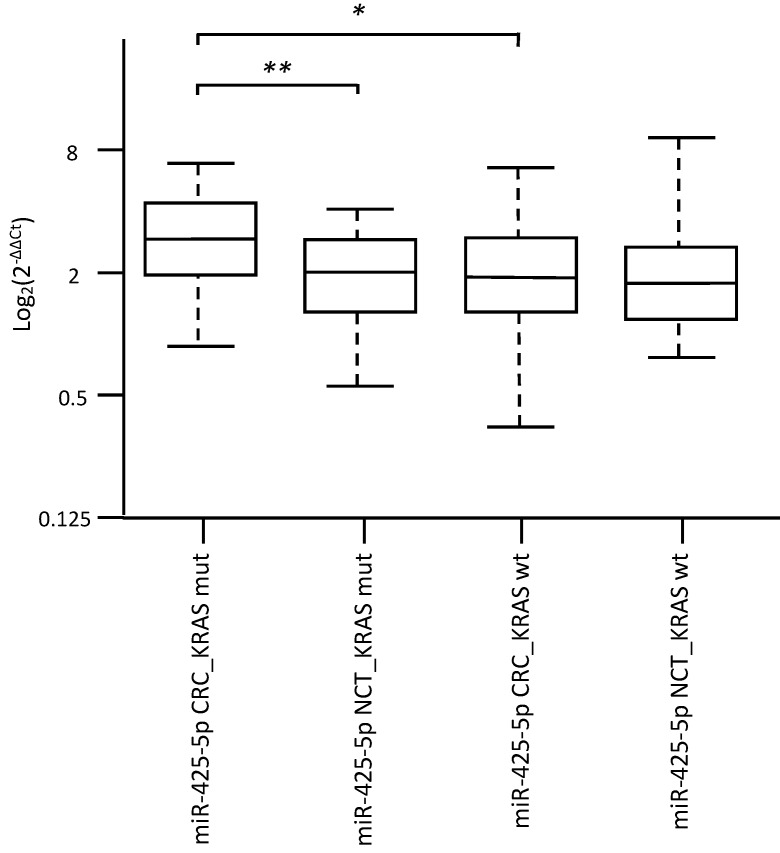
** Validation analysis for miR-425-5p expression levels by real-time polymerase chain reaction.** Box and whisker plots were used to summarize the distribution of miR-425-5p expression levels of 1.55 (interquartile range, 1.06-2.19) in KRAS-mutated CRC and 1.07 (interquartile range, 0.75-1.48) in their corresponding paired NCTs; 0.99 (interquartile range, 0.72-1.49) in KRAS-wildtype CRC and 0.96 (interquartile range, 0.69-1.31) in their corresponding paired NCTs. Statistical analysis by t-test showed significant differences with *p-value = 0.034 between KRAS-mutated CRC and KRAS-wildtype CRC, and with **p-value = 0.0008 between KRAS-mutated CRC and their corresponding paired NCTs. Additionally, t-test performed between KRAS-mutated CRC vs the entire cohort of NCT, showed significant differences with p-value = 0.009 (data not shown). Box plot explanation: upper horizontal line of box, 75th percentile; lower horizontal line of box, 25th percentile; horizontal bar within box, median; upper horizontal bar outside box, 90th percentile; lower horizontal bar outside box, 10th percentile. Circles represent outliers. The values of miR-425-5p expression levels are expressed as Log2 (2^-ΔΔCt^).

**Figure 3:**
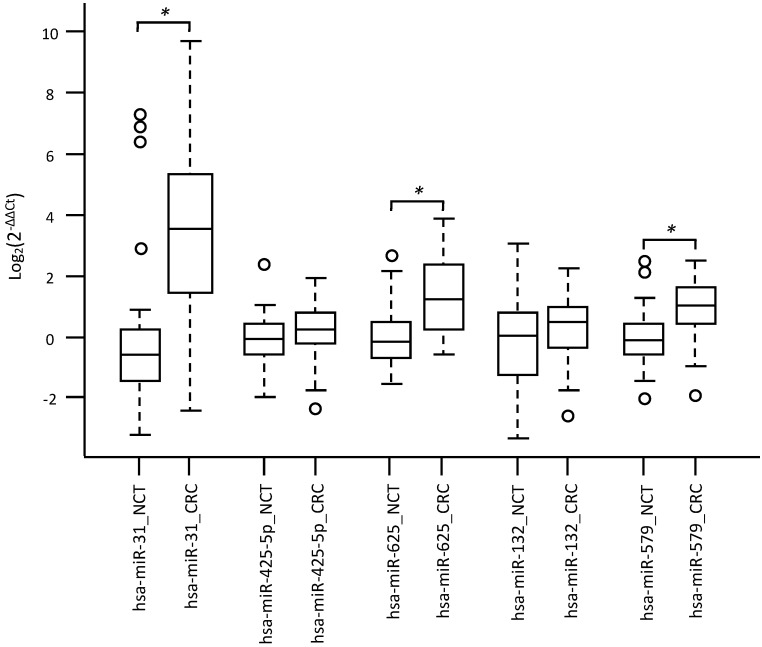
** Validation analysis for miR-31-5p, miR-425-5p, miR-625-5p, miR-132 and miR-579,** expression levels by RT-qPCR. Box and whisker plots were used to summarize the distribution of miR-31-5p expression levels of 11.12 (interquartile range, 3.18-37.93) in CRC and 0.65 (interquartile range, 0.38-1.16) in NCT; miR-425-5p expression levels of 1.19 (interquartile range, 0.86-1.75) in CRC and 0.94 (interquartile range, 0.68-1.35) in NCT; miR-625-5p expression levels of 2.31 (interquartile range, 1.14-5.24) in CRC and 0.89 (interquartile range, 0.62-1.39) in NCT; miR-132 expression levels of 1.39 (interquartile range, 0.85-1.96) in CRC and 1.03 (interquartile range, 0.41-1.71) in NCT; miR-579 expression levels of 1.98 (interquartile range, 1.35-3.01) in CRC and 0.91 (interquartile range, 0.70-1.31) in NCT. Statistical analysis by t-test showed significant differences for miR-31-5p with p-value = 2,59255E-08 between CRC and NCT; miR-625-5p with p-value = 1,93876E-06 between CRC and NCT; miR-579 with p-value = 3,13957E-05 between CRC and NCT. Box plot explanation: upper horizontal line of box, 75th percentile; lower horizontal line of box, 25th percentile; horizontal bar within box, median; upper horizontal bar outside box, 90th percentile; lower horizontal bar outside box, 10th percentile. Circles represent outliers. The values of all miRNAs expression levels are expressed as Log2 (2^-ΔΔCt^).

Specifically, overexpression of miR-31-5p and downregulation of miR-425-5p were found in KRAS-wildtype CRC compared to NCT. Overexpression of miR-625-5p was observed in KRAS-wildtype CRC respect to KRAS-mutated CRC and NCT. Downregulation of miR-579 and miR-132 were identified in KRAS-mutated CRC compared to KRAS-wildtype CRC and NCT. The differential expression determined by microarray analysis was validated by RT-qPCR for all five identified miRNAs. The RT-qPCR values did not confirm downregulation of miR-425-5p in KRAS-wildtype CRC compared to NCT, but strongly demonstrated that miR-425-5p was overexpressed in KRAS-mutated CRC compared to KRAS-wildtype CRC (p = 0.034) and to NCT (p = 0.0008) (Figure 2). RT-qPCR results showed no significant difference in expression of miR-31-5p, miR-625-5p and miR-579 comparing KRAS-mutated CRC and KRAS-wildtype CRC, but all miRNAs were showed overexpressed in CRC compared to NCT (p = 2.5E-08, p = 1.9E06, p = 3.1E05, respectively). No statistical evidence was found in the expression variations for miR-132 (Figure 3).

### Gene targets of miRNAs and functional analysis

**Figure 4:**
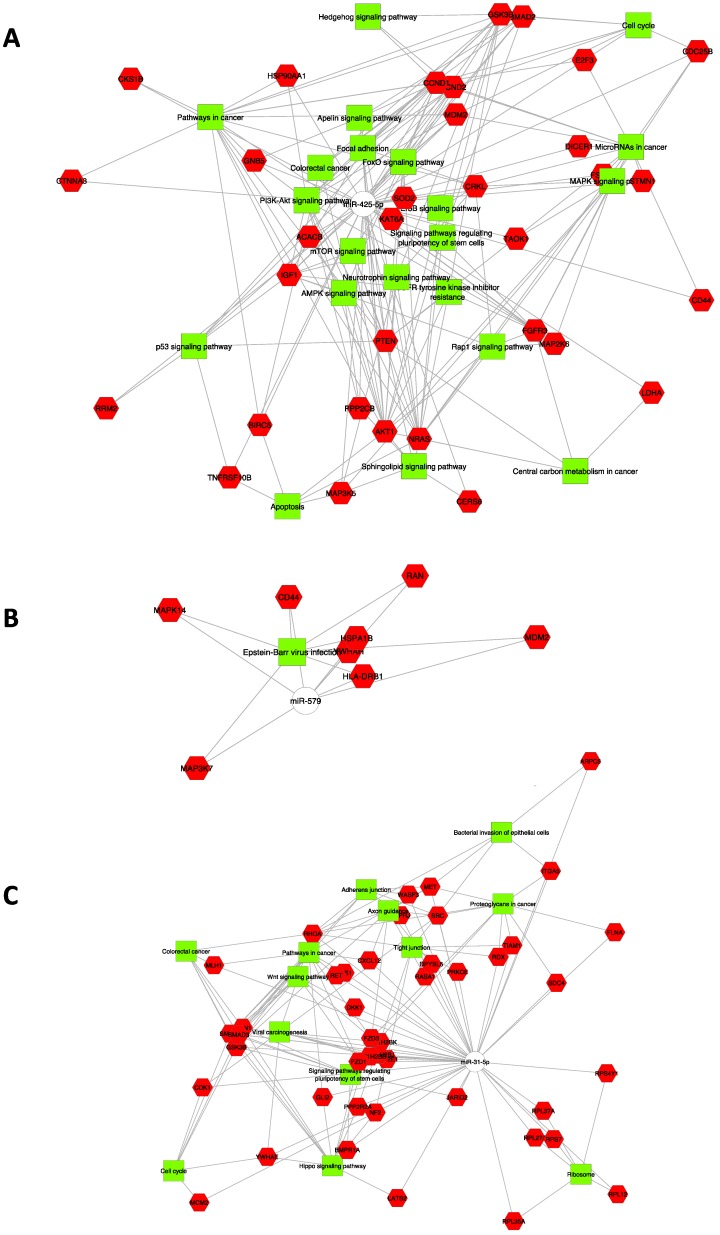
** Colorectal tumor interactome.** A: CRC interactome network developed using Cytoscape for 186 target genes of miRNA-425-5p; B: 137 target genes of miR-579; C: 175 target genes of miR-31-5p.

A functional enrichment analysis was performed on experimentally validated or *in silico* predicted targets of miR-425-5p, showing that it affects important biological processes. Significantly enriched gene sets include the following genes: AKT1, CDC25B, CRKL, FGFR3, MAP2K6, MAP3K5, NRAS, STMN1, TAOK1 that have key roles in MAPK signaling pathway; AKT1, CCND1, CCND2, FGFR3, GNB5, GSK3B, HSP90AA1, MDM2, NRAS, PPP2CB, PTEN all members of PI3K/AKT signaling pathway; CCND1, CCND2, IGF1, MDM2, PTEN, RRM2, TNFRSF10B involved in p53 signaling pathway, etc. (Figure 4A). Our *in silico* analysis has highlighted the importance of specific target genes for miR-425-5p related to apoptosis (TNFRSF10B, GSK3B, MAP2KE, MAP3K5 and TAOK1), required by the RNA interference and temporal RNA pathways to produce the small active RNA component that suppresses gene expression (DICER1 ribonuclease), and the MAP2KE, MAP3K5 and PP2CB genes, which exercise a negatively effect on cell growth.

CD44, HLA, RB1, HSPA1B, MAP3K7, MAPK14, MDM2, RAN, and YWHAH genes are members of Epstein-Barr virus infection pathway, identified as controlled by miR-579 (Figure 4B). Interestingly, miR-31-5p controls genes involved in pathways as well as Proteoglycans in cancer, Adherens and Tight junctions, Hippo signaling pathway, WNT signaling pathway, Colorectal cancer, Axon guidance, Cell Cycle, Viral carcinogenesis and Bacterial invasion of epithelial cells (Figure 4C).

Moreover, different miR-625-5p target genes were involved in biological process, such as negative regulation of phosphorylation and of MAPK cascade, positive regulation of signal transduction and peptidyl-amino acid modification (see Table S1).

### Association analysis of clinic-pathological and molecular features and miRNAs expression level

The clinico-pathologic features at diagnosis of these tumors are characterized by a median (IQR) time of survival of 31.5 (27.5-38.5) months. No patients died of other diseases or casualties. Thirty-five CRC patients were reported to be alive with no evidence of disease (NED) at the ended follow-up date.

The majority of the tumors has a right localization (24, 54.6%) and about half (20, 46.5%) were tumor stage III. The histologic grade was G2 in 68.2% of the cases and tumor infiltrating lymphocytes were present in the 31% of CRC samples. KRAS mutation was found in the 38.2%. MiR-31-5p, miR-579, and miR-625-5p were more frequently up-regulated (84.1%, 52.3%, and 52.3%, respectively), whereas miR-425-5p was down-regulated (81.4%).

Right localization of the tumor was more prevalent when miR-31-5p (p-value: 0.002) and miR-625-5p (p-value: 0.007) were up-regulated and miR-425-5p down-regulated (p-value: 0.02) (Table 1). On the other side, left localization was more frequent when miR-31-5p was down-regulated (p-value: 0.03). MiR-579 was found up-regulated in tumor stage I (p-value: 0.005).

The logistic regression analysis aimed to assess the relationship between miRNAs expression levels and clinico-pathological variables showed that miR-31-5p was significantly associated with a left localization of the tumor (OR: 0.1; p-value: 0.03), whereas miR-579 was significantly associated with tumor stage I (OR: 14.6; p-value: 0.02) (Table 2).

**Figure 5:**
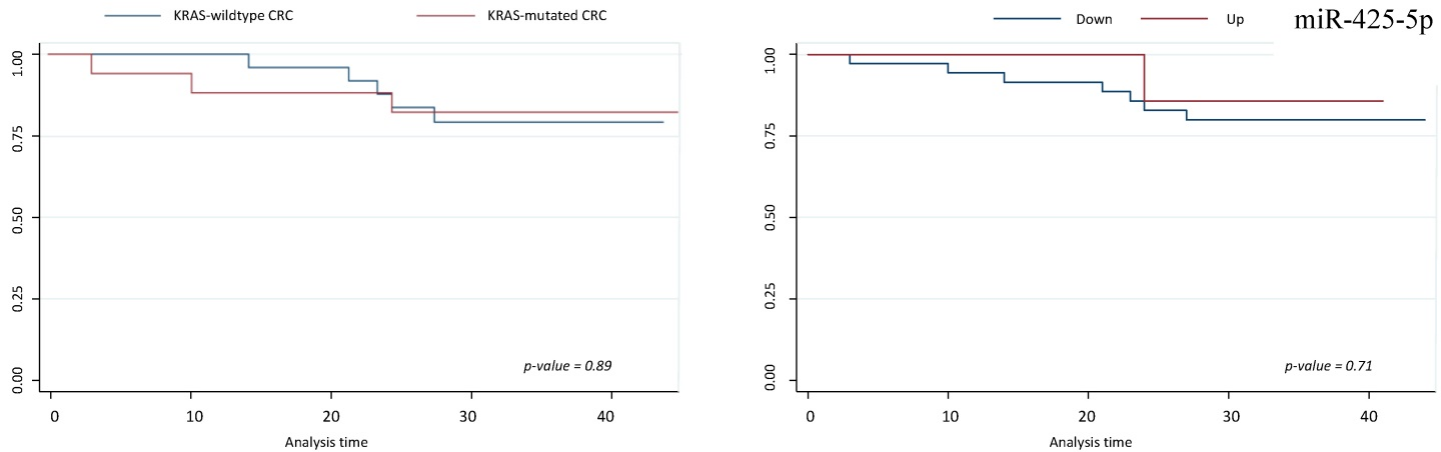
** Survival analysis for miR-425-5p.** On left, Kaplan-Meier analysis according to KRAS-mutated CRC vs KRAS-wildtype CRC; on right, Kaplan-Meier analysis according to up and down miR-425-5p expression level.

**Figure 6:**
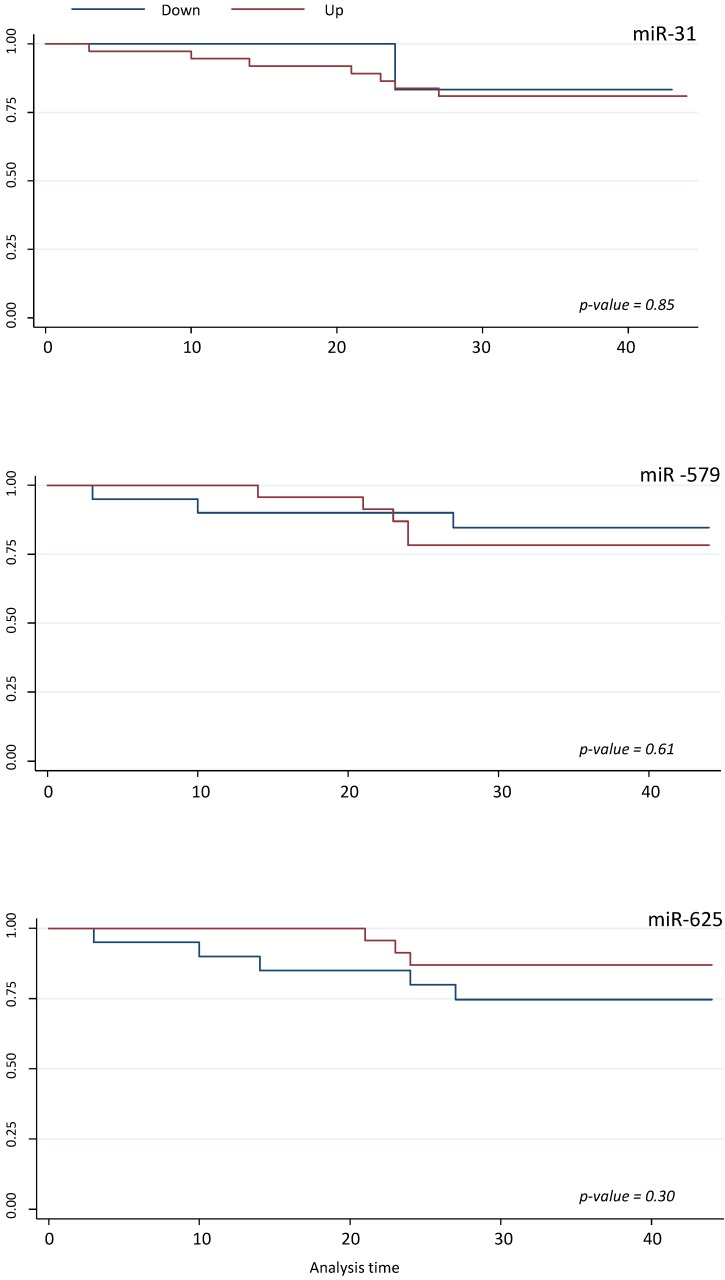
** Survival analyses for miR-31-5p, miR-625-5p, and miR-579.** Kaplan-Meier analysis according to up and down miR-31-5p, miR-625-5p, and miR-579 expression level.

Survival analyses did not show any statistically significant differences respect to miR-425-5p, miR-31-5p, miR-579 and miR-625-5p expression variations (Figures 5 and 6).

## Discussion

Our study confirmed a high percentage of KRAS missense somatic mutations (38.2%) in CRC samples, of which 94.5% of mutations were located in exon 2 and 5.5% in exon 3 of the KRAS gene, as evidenced in the literature. KRAS mutations were not identified on exon 4, codons 117 and 146, of KRAS gene, might be related to scare percentage of mutations, about 5% for two codons. The RASCAL study, which involved 2721 CRC patients from 13 different countries, have successfully clarified the role of KRAS mutations in the patient outcome and its poor prognostic significance 35. The RASCAL II study additionally proved that an increased risk of recurrence and/or death was associated to one mutation on codon 12, glycine to valine 35. In 2006, Lievre et al. reported for the first time the association between KRAS gene mutation and reduced response to anti-EGFR agents, proving that patients with KRAS wildtype genotype had the best overall survival 36. Therefore, RAS testing including KRAS exons 2, 3 and 4 (codons 12, 13, 59, 61, 117 and 146) is mandatory before treatment with anti-EGFR therapy in cancer patients 37. A better understanding of the CRC biology and establishing predictive factors are crucial for the identification of new therapeutic targets in CRC patients with KRAS-mutated gene to improve their outcome.

Our extensive analysis of miRNAs expression profile performed in tumor samples of CRC patients, has primarily detected an overexpression of miR-425-5p in KRAS-mutated CRC compared to KRAS-wild type CRC and NCT. Despite the fact that the exact functional significance of miR-425-5p is not known, there is evidence in literature that this miRNA may act as an oncogenetic gene in cancer pathogenesis. The miR-425-5p is involved in the progression of hepatocellular carcinoma 38, renal cell carcinoma 39, prostate carcinoma 40, cervical carcinoma 41 and gastric carcinoma 42.

Our *in silico* prediction demonstrates that miR-425-5p exerts regulatory effects on target genes involved in molecular networks, such as FOX-O, p53, PI3K-AKT, MAPK, Apelin and Hippo pathways, etc.. These pathways regulate cellular proliferation, migration/invasion, apoptosis and their deregulation contribute to the tumor development. These data could be correlated with the strong aggressiveness of KRAS-mutated CRC compared KRAS-wildtype CRC. Our data also contribute to supporting the correlation of different reporting patterns controlled by miR-425-5p with colorectal cancer pathogenesis 3,43,44.

Interesting target genes of miR-425-5p, which we identify, regulate cellular processes such as apoptosis, miRNAs production and inhibition of cellular growth. DICER1 is a key enzyme involved in the miRNA processing pathway that regulates mRNA-based gene silencing by the cleavage of miRNA precursor 45. A reduced expression of mRNA DICER1 is associated with an unfavorable prognosis in CRC patients 46 and the critical role of miRNAs in the process of reprogramming and determining a differentiated phenotype of CRC cells is blocked in the absence of DICER1 47. Finally, it has been identified that inflammation-induced JAK/STAT3 signaling leads to the development of CRC through proteasomal degradation of DICER1 by ubiquitin ligase complex of CUL4A(DCAF1) 48. Based on this assumption, we might propose that the restarted DICER1 protein function should contribute to reprogramming/reverting the neoplastic phenotype of CRC cells.

Several studies, supporting our data, have shown the importance of TNFRSF10B in TRAIL responsiveness in CRC 49,50. TNFRSF10B protein is a member of the TNF-receptor superfamily and contains an intracellular death domain. This receptor can be activated by ligand as TNFSF10/TRAIL/APO-2L transducing an apoptosis signal. Recently, Benoit et al. reported that treating the TRAIL-resistant HT29 and SW480 human cell lines with the PRC2 HMTase inhibitor 3-deazaneplanocin-A led to increased expression of the death receptor TNFRSF10B and consequently to cells apoptosis 51.

Results of this study provide evidence that some miR-425-5p targeted genes are involved in EGFR tyrosine kinase inhibitor resistance pathway, suggesting that miR-425-5p-based therapies might have a strong potential in targeting KRAS-driven CRC. This hypothesis is supported by the role of miR-425-5p overexpression in the modulation of CRC cells chemosensitivity to 5-fluorouracil and oxaliplatin treatments, depending on PDCD10 protein modulation by miR-425-5p. Moreover, high PTEN expression has been also reported to be associated with chemosensitivity to 5-fluorouracil and oxaliplatin treatments in CRC 52.

Our data proved that PDCD10 and PTEN proteins are involved in important cell biological process, such as positive regulation of kinase activity, transferase activity, and cell proliferation. PDCD10 protein alone controls the intrinsic apoptotic signaling pathway in response to oxidative stress. The overexpression of miR-425-5p in neoplastic cells might permit a failure modulation of these biological processes by downregulation of PDCD10 and PTEN proteins.

By comparing CRC vs NCT, we demonstrated a role in the oncogenesis of miR-31-5p, miR-625-5p and miR-579. Previous data on miR-31-5p showed strongly altered expression levels in different tumors. This miRNA could act as an oncogene 53-55 or alternatively as a suppressor gene depending on the different type of cancers 56-58. The acting mechanism of miR-31-5p is closely related to its target gene: in the same pathway, it causes different cellular effects, depending on the type of tumor, while it has similar functions in different organs if the target is the same 59.

We have proven that the target genes of miR-31-5p are involved in networks that control basic cell functions (growth, differentiation, apoptosis, etc.), and whose deregulation may explain the molecular mechanisms of tumorigenesis and the strong aggressiveness of CRC.

Based on our findings, we might assume that miR-31-5p contributes to CRC metastasis by looking at EMT, cell migration/invasion, vascular invasion by regulating the Hippo pathway, WNT pathway, Adherens and Tight junctions, and Signaling pathways regulating pluripotency of stem cells.

In the future, work will be done to characterize miR-31-5p and its signaling components by using them as informative clinical markers and promising therapeutic targets for treating cancer metastasis. Igarashi et al. stated that miR-31-5p overexpression, in absence of mutations in KRAS, NRAS and BRAF gene, was associated with short progression-free survival in patients with CRC treated with anti-EGFR therapies, suggesting that miR-31-5p could be a prognostic biomarker for anti-EGFR therapies 60.

The role of miR-579 in cancer cells is still poorly investigated, but this miRNA has been found overexpressed in CRC tissue and significantly associated with a poor overall or cancer-specific survival 61. The dysregulation of miR-579 in CRC with liver metastases suggests its involvement in tumor progression 62. In our study miR-579 was shown overexpressed in CRC, and CD44, HLA, RB1, HSPA1B, MAP3K7, MAPK14, MDM2, RAN and YWHAH, involved in Epstein-Barr virus infection pathway, were identified as its target genes. These genes need further studies to better decipher their role in the pathogenesis of CRC.

Our data identified, for the first time, an overexpression of miR-625-5p in CRC samples. The target genes of this miRNA are involved in the negative regulation of phosphorylation and of MAPK cascade, in the positive regulation of signal transduction and in the peptidyl-amino acid modification. Zhang et al. have reported that miR‐625‐5p negatively regulates melanoma glycolysis state by targeting PKM2 63. Upregulation of miR-625-5p was also identified in transient depletion of TIA-proteins in HeLa cells 64. Previous CRC studies have demonstrated deregulation of miR-625-3p associated with tumor metastasis and poor prognosis. Transfected miR‐625 mimics into HCT116 showed that miR‐625 can regulate CRC cell invasion and metastasis 65. Moreover, high expression of miR-625-3p has been associated with poor response to first-line oxaliplatin based-treatment of metastatic CRC patients 66.

Additionally, our data showed that overexpression of miR-31-5p and miR-625-5p combined with downregulation of miR-425-5p was characterized of the CRC localization on right site of colon, suggesting a distinct biology of CRC based on the site of tumors development (right, left or rectum, respectively). MiR-579 overexpression was found to correlate with tumor stage I, suggesting that its deregulation might play a major role in early pathogenesis of CRC and its progression. The limited cohort of our study does not have the necessary statistical power to highlight a prognostic significance of deregulation of miRNAs expression levels, and we could not demonstrate associations between deregulation of miRNAs expression and OS. Extensive and independent studies will be needed to identify the role of these miRNAs in the prognosis of CRC patients.

In conclusion, our results showed that miR-425-5p might act as an oncogene to participate in the pathogenesis of KRAS-mutated CRC and contribute to increase the aggressiveness of these subcategories of CRC, controlling a complex molecular network. Our study implies clinical applications, based on the involvement of miR-425-5p targeted genes in EGFR tyrosine kinase inhibitor resistance pathway, suggesting that miR-425-5p overexpression might represents in CRC an epigenetic mechanism of resistance to anti-EGFR therapies. MiR-425-5p-based treatments might have a strong potential in targeting KRAS-driven CRC to overcome the drug resistance and to dissect and control the molecular network related to cancer progression. Based on our results, we propose the possible use of miR-425-5p as a stool and/or blood-based biomarker, which could represent reliable and non-invasive methods of categorizing poor prognostic KRAS-mutated CRC patients.

## Supplementary Material

Supplementary tables.

## Figures and Tables

**Table 1:** Clinico-pathological and molecular data of CRC patients according to miRNAs expression levels deregulation.

Variables		miR-31-5p			miR-625-5p			miR-579			miR-425-5p		
		Down	Up	p-value*	Down	Up	p-value*	Down	Up	p-value*	Down	Up	p-value*
*Mortality, n (%)*		2 (28.6)	7 (18.9)	0.62	6 (28.6)	3 (13.0)	0.27	4 (19.1)	5 (21.7)	1.0	7 (20.0)	2 (25.0)	1.0
*Median (IQR)*		37 (24-39)	31 (28-37)	0.74	31 (26-37)	33 (28-39)	0.28	35 (28-38)	29 (26-39)	0.32	32 (28-39)	28.5 (25-36)	0.34
*Localitation, n (%)*	*Right*	0 (0.0)	24 (64.9)	0.002	7 (33.3)	17 (73.9)	0.007	12 (57.1)	12 (52.2)	0.74	22 (62.9)	1 (12.5)	0.02
	*Left*	5 (71.4)	9 (24.3)	0.03	9 (42.9)	4 (17.4)	0.10	8 (38.1)	5 (21.7)	0.33	9 (25.7)	5 (62.5)	0.09
	*Rectum*	2 (28.6)	4 (10.8)	0.24	5 (23.8)	2 (8.7)	0.23	1 (4.8)	6 (26.1)	0.10	4 (11.4)	2 (25.0)	0.31
*Tumor stage, n (%)*	*I*	2 (28.6)	9 (25.0)	1.0	4 (20.0)	7 (30.4)	0.50	1 (5.0)	10 (43.5)	0.005	9 (26.5)	2 (25.0)	1.0
	*II*	2 (28.6)	4 (11.1)	0.25	4 (20.0)	3 (13.0)	0.69	3 (15.0)	4 (17.4)	1.0	4 (11.8)	2 (25.0)	0.32
	*III*	2 (28.6)	18 (50.0)	0.42	8 (40.0)	11 (47.8)	0.61	12 (60.0)	7 (30.4)	0.07	16 (47.1)	3 (37.5)	0.71
	*IV*	1 (14.3)	5 (13.9)	1.0	4 (20.0)	2 (8.7)	0.39	4 (20.0)	2 (8.7)	0.39	5 (14.7)	1 (12.5)	1.0
*Histologic grade*	*G1*	0 (0.0)	2 (5.4)	0.53	1 (4.8)	1 (4.4)	1.0	1 (4.8)	1 (4.4)	1.0	1 (2.9)	1 (12.5)	0.34
	*G2*	6 (85.7)	24 (64.9)	0.40	16 (76.2)	15 (65.2)	0.43	14 (66.7)	17 (73.9)	0.74	25 (71.4)	5 (62.5)	0.68
	*G3*	1 (14.3)	11 (29.7)	0.65	4 (19.1)	7 (30.4)	0.49	6 (28.6)	5 (21.7)	0.73	9 (25.7)	2 (25.0)	1.0
*Tumor infiltrating lymphocytes, n (%)*		2 (28.6)	11 (31.4)	1.0	6 (31.6)	7 (30.4)	1.0	3 (15.8)	10 (43.5)	0.09	11 (33.3)	2 (25.0)	1.0
*KRAS mutational status, n (%)*		4 (57.1)	13 (35.1)	0.40	7 (33.3)	10 (43.5)	0.49	9 (42.9)	8 (34.8)	0.58	11 (31.4)	5 (62.5)	0.13

n = number IQR = interquartile range * The p-values are bold where they are less than or equal to the significance level of 0.05.

**Table 2:** Univariate analysis to assess the relationship between miRNAs expression levels deregulation and clinico-pathological features

Variables		miR-31-5p		miR-625-5p		miR-579		miR-425-5p	
		OR (95% CI)	p-value*	OR (95% CI)	p-value	OR (95% CI)	p-value*	OR (95% CI)	p-value
*Localitation*	*Right*	-	-	5.6 (1.5-20.8)	0.009	0.8 (0.2-2.7)	0.74	0.1 (0.0-0.8)	0.03
	*Left*	0.1 (0.0-0.8)	0.03	0.3 (0.1-1.1)	0.07	0.5 (0.1-1.7)	0.24	4.8 (1.0-24.3)	0.06
	*Rectum*	0.3 (0.0-2.1)	0.23	0.3 (0.1-1.8)	0.19	7.1 (0.8-64.6)	0.08	2.6 (0.4-17.4)	0.33
*Tumor stage (I-IV)*		1.3 (0.6-2.8)	0.57	0.8 (0.4-1.4)	0.42	0.4 (0.2-0.8)	0.007	0.9 (0.4-1.9)	0.76
*Tumor stage*	*I*	0.8 (0.1-5.1)	0.84	1.8 (0.4-7.2)	0.44	14.6 (1.7-128.4)	0.02	0.9 (0.2-5.5)	0.93
	*II*	0.3 (0.1-2.2)	0.24	0.6 (0.1-3.1)	0.54	1.2 (0.2-6.1)	0.83	2.5 (0.4-16.9)	0.35
	*III*	2.5 (0.4-14.6)	0.31	1.4 (0.4-4.6)	0.61	0.3 (0.1-1.0)	0.06	0.7 (0.1-3.3)	0.63
	*IV*	1.0 (0.1-9.8)	0.98	0.4 (0.1-2.3)	0.30	0.4 (01-2.4)	0.30	0.8 (0.1-8.3)	0.87
*Histologic grade (G1-G3)*		1.5 (0.3-7.3)	0.64	1.6 (0.5-5.3)	0.44	0.8 (0.2-2.5)	0.67	0.7 (0.1-3.1)	0.61
*Histologic grade*	*G1*	-	-	0.9 (0.1-15.5)	0.95	0.9 (0.1-15.5)	0.95	4.9 (0.3-87.3)	0.28
	*G2*	0.3 (0.0-2.8)	0.30	0.6 (0.2-2.2)	0.43	1.4 (0.4-5.2)	0.59	0.7 (0.1-3.3)	0.62
	*G3*	2.5 (0.3-23.6)	0.41	1.9 (0.5-7.6)	0.39	0.7 (0.1-2.7)	0.60	1.0 (0.2-5.7)	0.97
*Tumor infiltrating lymphocytes*		1.2 (0.2-6.9)	0.88	1.0 (0.3-3.5)	0.94	4.1 (0.9-18.1)	0.06	0.7 (0.1-3.9)	0.65
*KRAS mutational status*		0.4 (0.1-2.1)	0.28	1.5 (0.5-5.2)	0.49	0.7 (0.2-2.4)	0.58	3.6 (0.7-18.0)	0.11

OR: Odds Ratio; CI: Confidence Interval * The p-values are bold where they are less than or equal to the significance level of 0.

## Abbreviations

CRC: Colorectal Cancer
NCT: Normal Colon Tissues
miRNA: microRNA
WHO: World Health Organization
RIN: RNA Integrity Number
RT-qPCR: Quantitative-Real Time-PCR
GO: Gene Ontology
KEGG: Kyoto Encyclopedia of Genes and Genomes
FDR: False Discovery Rate
SAM: Statistical analysis of microarray
TLDA: TaqMan Low Density Array
IQR: Interquartile range
NED: No evidence of disease
EMT: Epithelial-Mesenchymal Transition
has: *Homo Sapiens*
mmu-miR: Mus Musculus miRNA
